# T2 Toxin-Induced Changes in Cocaine- and Amphetamine-Regulated Transcript (CART)-Like Immunoreactivity in the Enteric Nervous System Within Selected Fragments of the Porcine Digestive Tract

**DOI:** 10.1007/s12640-016-9675-8

**Published:** 2016-10-13

**Authors:** Krystyna Makowska, Slawomir Gonkowski, Lukasz Zielonka, Michal Dabrowski, Jaroslaw Calka

**Affiliations:** 1 Department of Clinical Physiology, Faculty of Veterinary Medicine, University of Warmia and Mazury in Olsztyn, ul. Oczapowskiego 13, 10-718 Olsztyn, Poland; 2 Department of Veterinary Prevention and Feed Hygiene, Faculty of Veterinary Medicine, University of Warmia and Mazury in Olsztyn, ul. Oczapowskiego 13, 10-718 Olsztyn, Poland

**Keywords:** Mycotoxin, T-2 toxin, Cocaine- and amphetamine- regulated transcript (CART), Enteric nervous system (ENS), Pig

## Abstract

T-2 toxin is a mycotoxin produced by some Fusarium species, which may affect the synthesis of DNA and RNA and causes various pathological processes. Till now, the influence of T-2 toxin on the enteric nervous system (ENS) located in the wall of gastrointestinal tract has not been studied. On the other hand, cocaine- and amphetamine-regulated transcript (CART) is one of enteric neuronal factors, whose exact functions in the intestines still remain not fully explained. The present study describes the influence of low doses of T-2 toxin on CART-positive neuronal structures in porcine stomach, duodenum, and descending colon. Distribution of CART was studied using the double immunofluorescence technique in the plexuses of the ENS, as well as in nerve fibers within the circular muscle and mucosal layers of porcine gastrointestinal tract. Generally, after T-2 toxin administration the greater number of CART-LI structures were studied, but intensity of changes depended on part of the ENS and digestive tract fragment studied. The obtained results show that even low doses of T-2 toxin may change the expression of CART in the ENS.

## Introduction

**Fig. 1:**
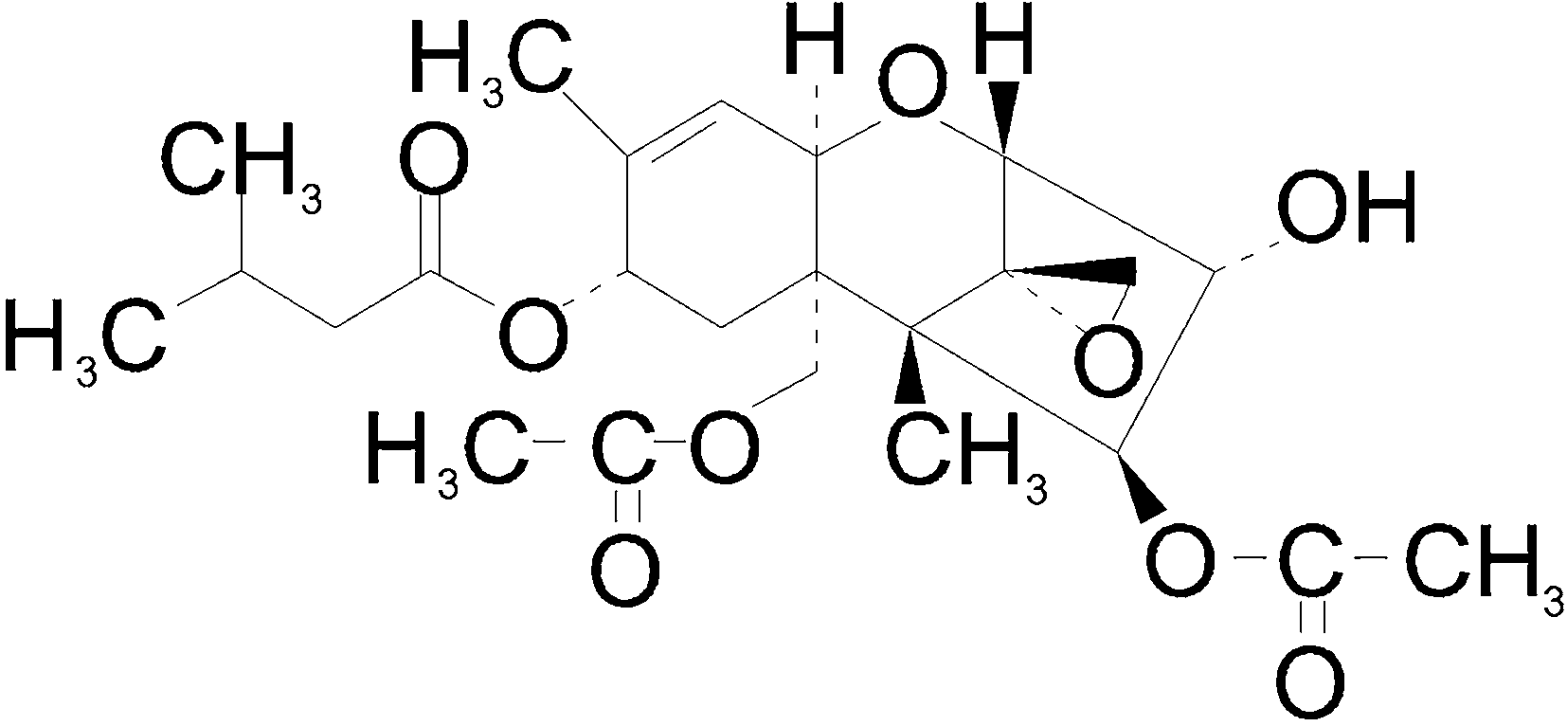
Scheme of the chemical structure of T-2 toxin molecule

Mycotoxins, naturally produced by some representatives of the fungi kingdom, are very numerous and differential group of substances, which often can contaminate the food and may have negative impact on living organisms. One of the mycotoxins that is important for the human and animal health situation is T-2 toxin, structurally belonging to chemical substances called trichothecenes (Fig. 1), which are characterized by a tetracyclic sesquiterpenoid ring system (Marin et al. 2013). This substance is synthetized by some *Fusarium* species (primarily by *F. sporotrichioides*, *F. langsethiae*, *F. acuminatum*, and *F. poae*) and it has been described in all most often cultivated over the world cereal species, such as wheat, oats, barley, and corn, as well as in foodstuffs produced from the above-mentioned grains (De Ruyck et al. 2015). Till now, various types of negative influence of T-2 toxin on living organisms have been described. First of all, longer exposition to this toxin can contribute to the development of alimentary toxic aleukia (ATA), which is characterized by various intestinal and general symptoms including vomiting, nausea, diarrhea, leukopenia, high fever, as well as inflammatory processes within the skin and in some cases leading to death (Lutsky and Mor 1981; De Ruyck et al. 2015). Moreover, T-2 toxin is known as the factor, which can influence on functions of thymus, spleen, and, on grounds of the ability to the crossing of blood–brain barrier, on nervous system (Martin et al. 1986; Doi and Uetsuka 2011; Weidner et al. 2013; Agrawal et al. 2015). At the cellular scale, T-2 toxin activity relies on the damage of mitochondria, inhibition of DNA and RNA synthesis, as well as suppression of apoptosis (Marin et al. 2013; De Ruyck et al. 2015).

**Fig. 2:**
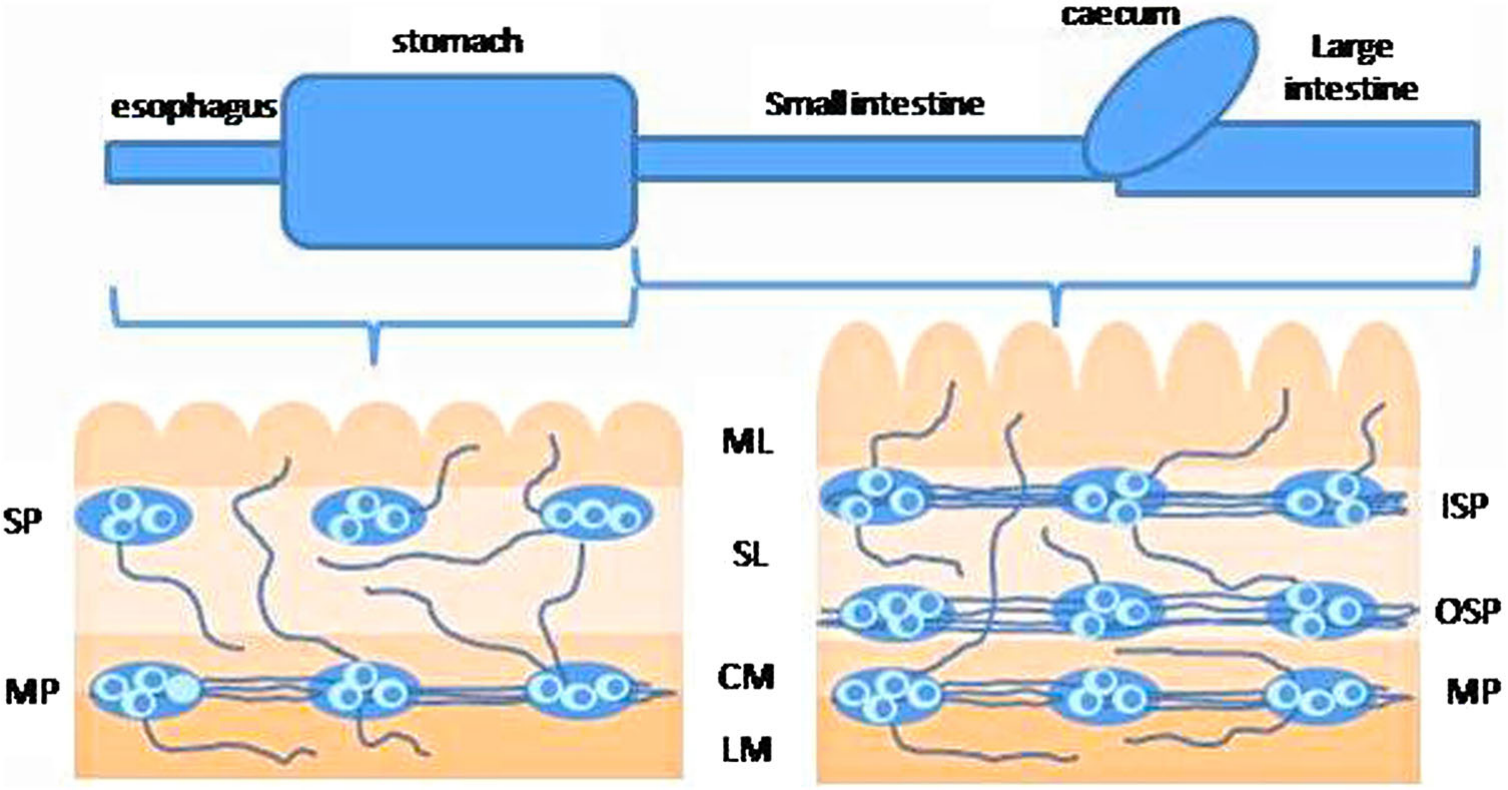
Scheme of the enteric nervous system in the porcine gastrointestinal tract. Elements of the wall of GI tract: *LM* longitudinal muscle layer, *CM* circular muscle layer, *SL* submucosal layer, *ML* mucosal layer. Elements of the enteric nervous system: *MP* myenteric plexus, *SP* gastric submucous plexus; *OSP* intestinal outer submucous plexus; *ISP* intestinal inner submucous plexus

In spite of the relatively well-known results of T-2 toxin poisoning, many aspects of its actions (especially in low doses) are completely unknown. One of them is the influence on the enteric nervous system (ENS), which is located in the wall of the digestive tract and on the grounds of complex building, high number of neurons and considerable independence from the central nervous system is often called the “second” or “intestinal brain” (Furness et al. 2014). In big mammals, the anatomy of the ENS depends on the segment of digestive tract (Fig. 2). Namely, in the stomach it consists of two intramural ganglionated plexuses: myenteric plexus (MP) located between longitudinal and circular muscular layers and submucous plexus (SP)—near the lamina propria of the mucosa. In the small and large intestine, submucous plexus undergoes a division to outer submucous plexus (OSP) located near internal side of the circular muscle layer and inner submucous plexus (ISP)—positioned in the same place like submucous plexus in the stomach (Gonkowski 2013; Bulc et al. 2015; Rekawek et al. 2015).

It is known that ENS responds to various pathological factors, including inflammatory processes, bacterial infections, toxins, and extra-intestinal diseases by structural, functional, and neurochemical changes (Vasina et al. 2006). The most visible are fluctuations in the expression of neuromediators and/or neuromodulators, which manifest adaptive and/or neuroprotective processes within enteric neurons under acting stimuli (Gonkowski et al. 2003; Vasina et al. 2006; Gonkowski 2013).

One of several dozen neuronal active substances, which till now have been described in the ENS, is cocaine- and amphetamine-regulated transcript peptide (CART). For the first time, CART was described in 1981 (Spiess et al. 1981) and since then it has been noted in the ENS of many species, including human (Ellis and Mawe 2003; Ekblad 2006; Arciszewski et al. 2009; Gonkowski et al. 2012; Bulc et al. 2014). Nonetheless, exact functions of this peptide in the gastrointestinal tract both in physiological conditions and during pathological processes are still controversial and not fully explained.

So, the aim of this study was to describe for the first time the influence of low doses (suggested permissible level in feed for farming animals) of T-2 toxin on CART-like immunoreactivity within the ENS of selected parts of digestive tract in pig, which currently is considered to be an optimal laboratory animal (much better then rodents) to simulation of processes in the human gastrointestinal tract due to anatomical and physiological resemblances in the ENS between these two species (Verma et al. 2011).

## Materials and Methods

### Tissue Preparation

Ten immature gilts (8 weeks of age, about 18 kg body weight) of the large White polish breed were used during the present experiment. The animals were divided into two groups: control (C group) and experimental (T-2 group), each of which consisted of five pigs. Control animals received empty gelatin capsules, once daily before the morning feeding for 42 days. In the event of experimental pigs, capsules were filled with T-2 toxin with a dose of 200 μg/kg of feed (suggested permissible level in feed for pig which was proposed as the lowest level by Eriksen and Pettersson 2004). During experiment, animals were kept in standard conditions, and all experimental actions were made in accordance with instructions of Local Ethical Committee for Animal Experiments in Olsztyn (Poland) (decision from Nov 28, 2012, identification code 73/2012/DTN).

After experimental period, all pigs were euthanized using an overdose of sodium thiopental (Thiopental, Sandoz, Kundl-Rakúsko, Austria) and fragments of the stomach, duodenum, and descending colon were collected immediately after death of animals. Tissues were sampled from the selfsame fragments of particular segments of the gastrointestinal tract. In the case of stomach, the sample of 3 cm^2^ came from the ventral part of corpus, in the central line, 20 cm from the cardia. From intestines, samples (2 cm long) were taken from duodenum (5 cm from the pylorus) and descending colon (from the part contiguous with inferior mesenteric ganglia). Immediately after collecting, samples were fixed in a solution of 4 % buffered paraformaldehyde (pH 7.4) for 1 h, rinsed in phosphate buffer (0.1 M, pH 7.4, at 4 °C) for 3 days with the exchange of buffer every day, and inserted into 18 % phosphate buffered sucrose (at 4 °C) for 2 weeks. Then samples were frozen at −22 °C, cut perpendicular to the lumen of the GI tract into 14-μm-thick sections using microtome (Microm, HM 525, Walldorf, Germany), and fixed on glass slides.

### Immunofluorescence Procedure

The slices of stomach and intestine were examined using standard double-labeling immunofluorescence technique that has been described previously by Wojtkiewicz et al. (2013). In short, it was as follows: after drying (45 min. at room temperature) and incubation with blocking solution containing 10 % goat serum, 0.1 % bovine serum albumin (BSA), 0.01 % NaN_3_, Triton X-100, and thimerosal in buffered NaCl solution (PBS) (1 h, room temp.), the samples were treated with the combination of antisera directed towards protein gene-product 9.5 (PGP 9.5; mouse monoclonal, Biogenesis, UK, working dilution 1:1000, used here as a pan neuronal marker) and CART (rabbit monoclonal, Phoenix Pharmaceuticals, USA, 1:16,000) and incubated overnight in a humidity chamber at room temperature. Then probes were incubated with the combination of species-specific secondary antibodies, i.e., Alexa fluor 488 donkey anti-mouse IgG and Alexa fluor 546 donkey anti-rabbit IgG, both from Invitrogen, Carlsbad, CA, USA, working dilution 1:1000 (1 h, room temp.). The rinsings of probes in buffered NaCl solution (PBS, 0.1 mol, pH 7.4, 15 min × 3) were made between each step of the above-mentioned procedure.

Specificity of the labeling was verified by standard control procedures, including pre-absorption, omission, and replacement tests. The pre-absorption consisted in replacement active primary antibodies by the same antibodies, but pre-absorbed with native human protein gene-product 9.5 (AbD Serotec, UK) and synthetic CART peptide (Phoenix Pharmaceuticals, USA) at a concentration of 20 µg/ml for 18 h, at 37 °C. These procedures eliminated specific stainings.

Immunostained tissue slices were evaluated using Olympus BX51 microscope equipped with epi-fluorescence and appropriate filter sets. To calculate the percentage of CART-like immunoreactive (CART-LI) neurons, at least 500 cell bodies immunoreactive to PGP 9.5 in particular types of enteric plexuses (MP, OSP and ISP) in each animal were evaluated in terms of CART-like immunoreactivity and only cells with well-visible nucleus were considered during experiment. The obtained results were presented as mean ± SEM.

Moreover, during the present study two methods were used to define the density of CART-LI nerve fibers. In case of intraganglionic nerve processes, the indication of this value was based on arbitrary cautioning scale from (−) (the absence of studied fibers) to (++++) (very frequent system of studied fibers). In contrast, CART-positive nerves in the muscular and mucosal layers were evaluated per observation field (0.1 mm^2^). Nerve fibers were counted in four tissue sections per animal (in five field per section) and the obtained data were pooled and presented as mean ± SEM. To prevent double counting of neuronal cells and nerve fibers, the evaluated sections of the GI tract were located at least 150 µm apart. Statistical analysis was made with Anova-test (Statistica 9.1, StatSoft, Inc.) and differences were considered statistically significant at *p* ≤ 0.05.

## Results

During the present study, CART-LI neuronal structures have been noted in the enteric nervous system of the stomach, duodenum, and descending colon in both control animals and in pigs after T-2 toxin administration. Their number clearly depended on the “type” of enteric ganglion, as well as the segment of the GI tract studied (Table 1).

**Table 1:** CART peptide-like immunoreactive (CART-LI) perikarya and nerve fibers in porcine stomach, duodenum, and descending colon under physiological conditions (C group) and after T-2 toxin administration (T2 group)

Stomach	C group	T2 group
CML^a^	13.36 ± 0.75	20.25 ± 1.32
MP		
CB^b^	46.24 ± 2.12	63.59 ± 1.12
NF^c^	+	++
SP		
CB^b^	6.39 ± 0.17	16.33 ± 1.30
NF^c^	+	+
S/ML^a^	0.83 ± 0.25	2.92 ± 0.45
Duodenum		
CML^a^	15.99 ± 0.80	26.66 ± 1.73
MP		
CB^b^	38.10 ± 3.43	63.80 ± 0.83
NF^c^	+++	+++
OSP		
CB^b^	28.70 ± 0.90	53.31 ± 1.49
NF^c^	++	++
ISP		
CB^b^	21.96 ± 1.85	35.86 ± 1.85
NF^c^	++	+++
S/ML	3.07 ± 0.14	8.3 ± 1.18
Descending colon		
CML^a^	15.33 ± 1.77	23.37 ± 1.04
MP		
CB^b^	33.43 ± 3.39	47.16 ± 1.30
NF^c^	+++	+++
OSP		
CB^b^	27.50 ± 1.07	41.38 ± 1.79
NF^c^	++	+++
ISP		
CB^b^	19.07 ± 4.11	42.35 ± 6.18
NF^c^	+	++
S/ML^a^	1.94 ± 0.35	3.75 ± 0.33

Differences between C group and T2 group in all parts of gastrointestinal tract studied are statistically significant (*p* ≤ 0.05)

*CML* circular muscle layer, *MP* myenteric plexus, *SP* gastric submucous plexus, *OSP* intestinal outer submucous plexus, *ISP* intestinal inner submucous plexus, *S/ML* submucosal/mucosal layer, *CB* cell bodies, *NF* nerve fibers

^a^Average number of nerve profiles per area studied (mean ± SEM)

^b^Relative frequency of particular neuronal subclasses in presented as  % (mean ± SEM) of all neurons counted within the ganglia stained for PGP 9.5 (used as pan neuronal marker). At least 500 PGP 9.5-positive cell bodies were evaluated in particular types of enteric plexuses in each animal

^c^The density of intraganglionic nerve fibers positive for CART is presented in arbitrary units

**Fig. 3:**
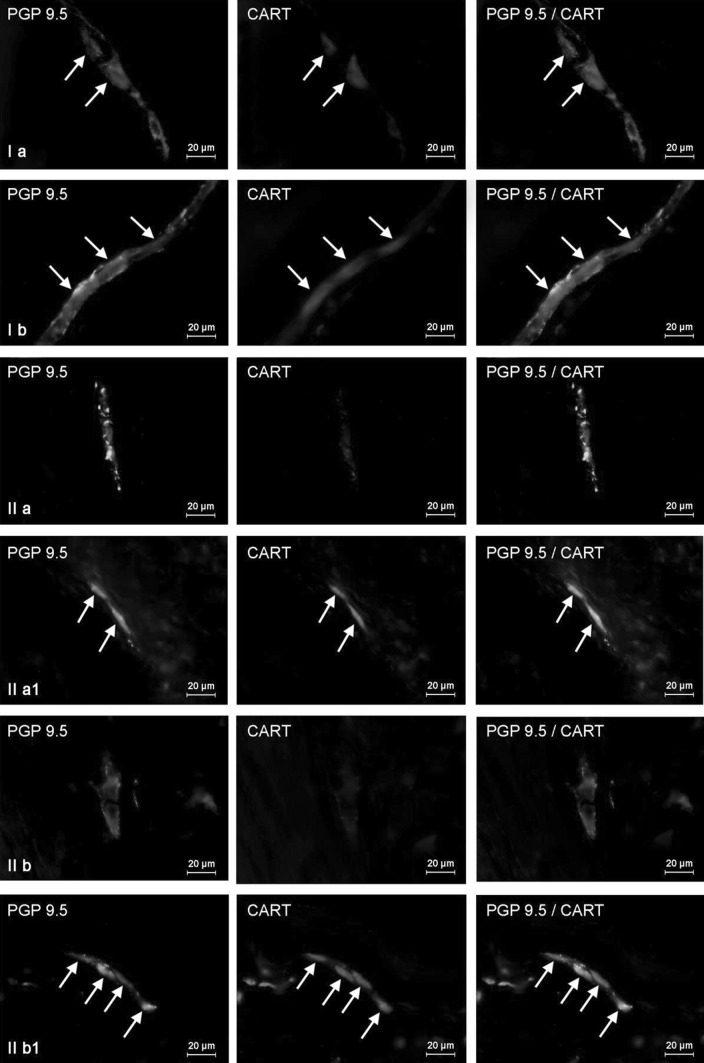
Distribution pattern of nervous structures immunoreactive to protein gene-product 9.5 (PGP 9.5)—used as pan neuronal marker and CART in the wall of porcine stomach under physiological conditions (**a**, **a1**) and after T2-toxin administration (**b**, **b1**); **I** myenteric plexus, **II** two kinds of submucous plexus. CART-positive neurons are indicated by arrows. The right column of the pictures shows the overlap of both stainings

**Fig. 4:**
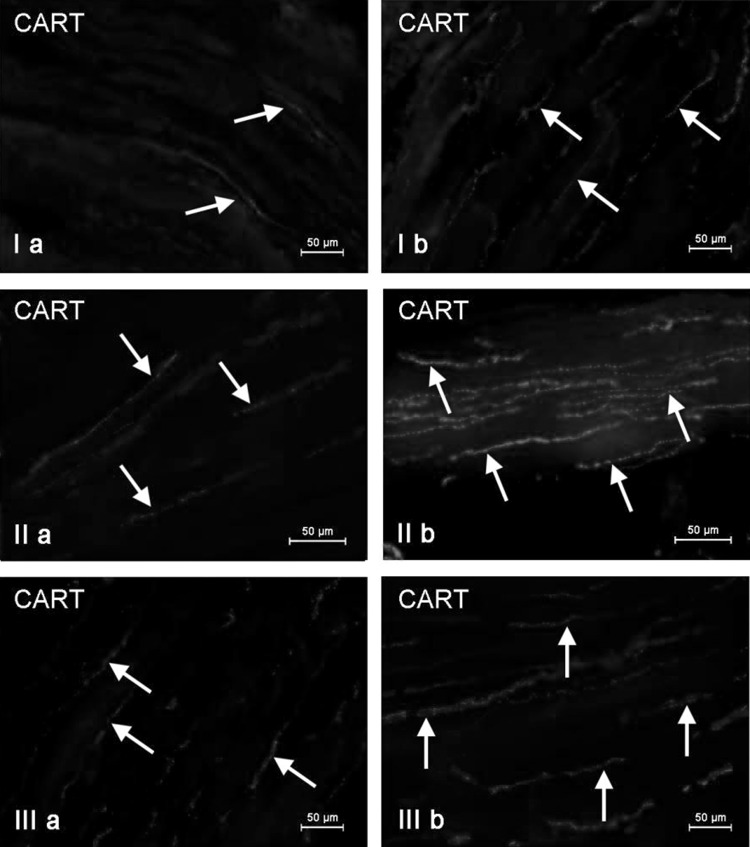
Nerve fibers immunoreactive to CART in the circular muscle layer of the porcine stomach (**I**), duodenum (**II**), and descending colon (**III**) under physiological conditions (**a**) and after T-2 toxin administration (**b**)

**Fig. 5:**
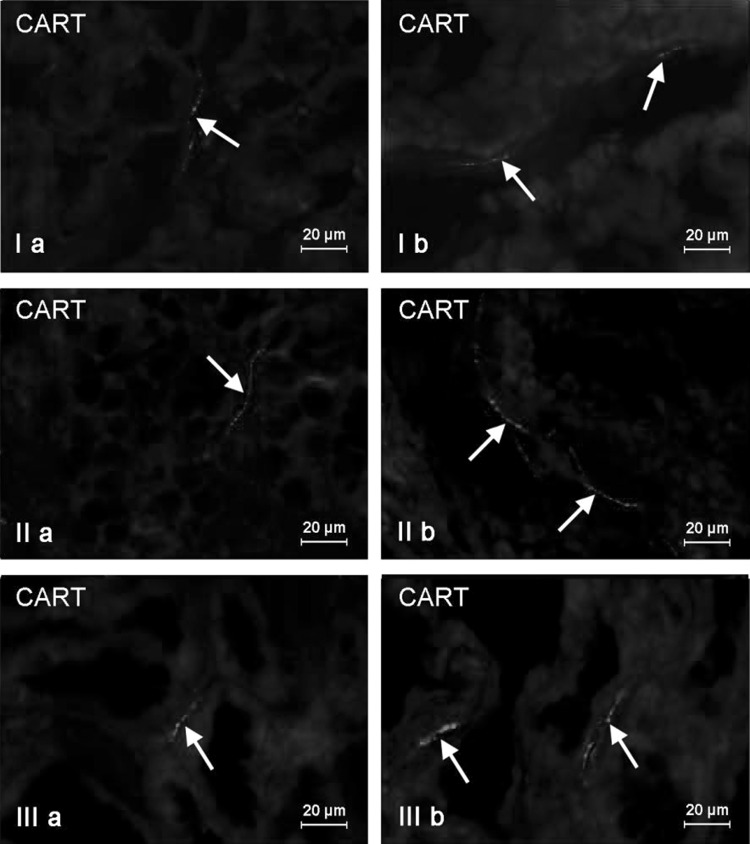
Nerve fibers immunoreactive to CART in the mucous layer of the porcine stomach (**I**), duodenum (**II**), and descending colon (**III**) under physiological conditions (**a**) and after T-2 toxin administration (**b**)

In the stomach of control animals, the majority of CART-positive neurons have been observed within myenteric plexus, where such cells amounted to almost half (46.24 ± 2.12 %) of all neurons immunoreactive to neuronal marker—PGP 9.5 (Fig. 3Ia). They were accompanied by single (+) intraganglionic nerves immunoreactive to CART. In contrast, small percentage of CART-positive neuronal cells (6.39 ± 0.17 % of all neurons) was investigated in the gastric submucous plexuses (Table 1). Moreover, two “kinds” of ganglia in this plexus were observed. The majority of them have no CART-LI neurons (Fig. 3IIa), but in some submucosal ganglia even all neurons were immunoreactive to this peptide (Fig. 3IIa1). The density of intraganglionic CART-positive nerves in gastric SP was similar to those observed in MP (+). Moreover, under physiological conditions, a density network of CART-positive nerves was observed within the gastric muscular layer (an average of 13.36 ± 0.75 fibers per observation field) (Fig. 4Ia), while in the mucosa of the stomach only single such nerves was investigated (0.83 ± 0.25 per observation field) (Fig. 5Ia).

**Fig. 6:**
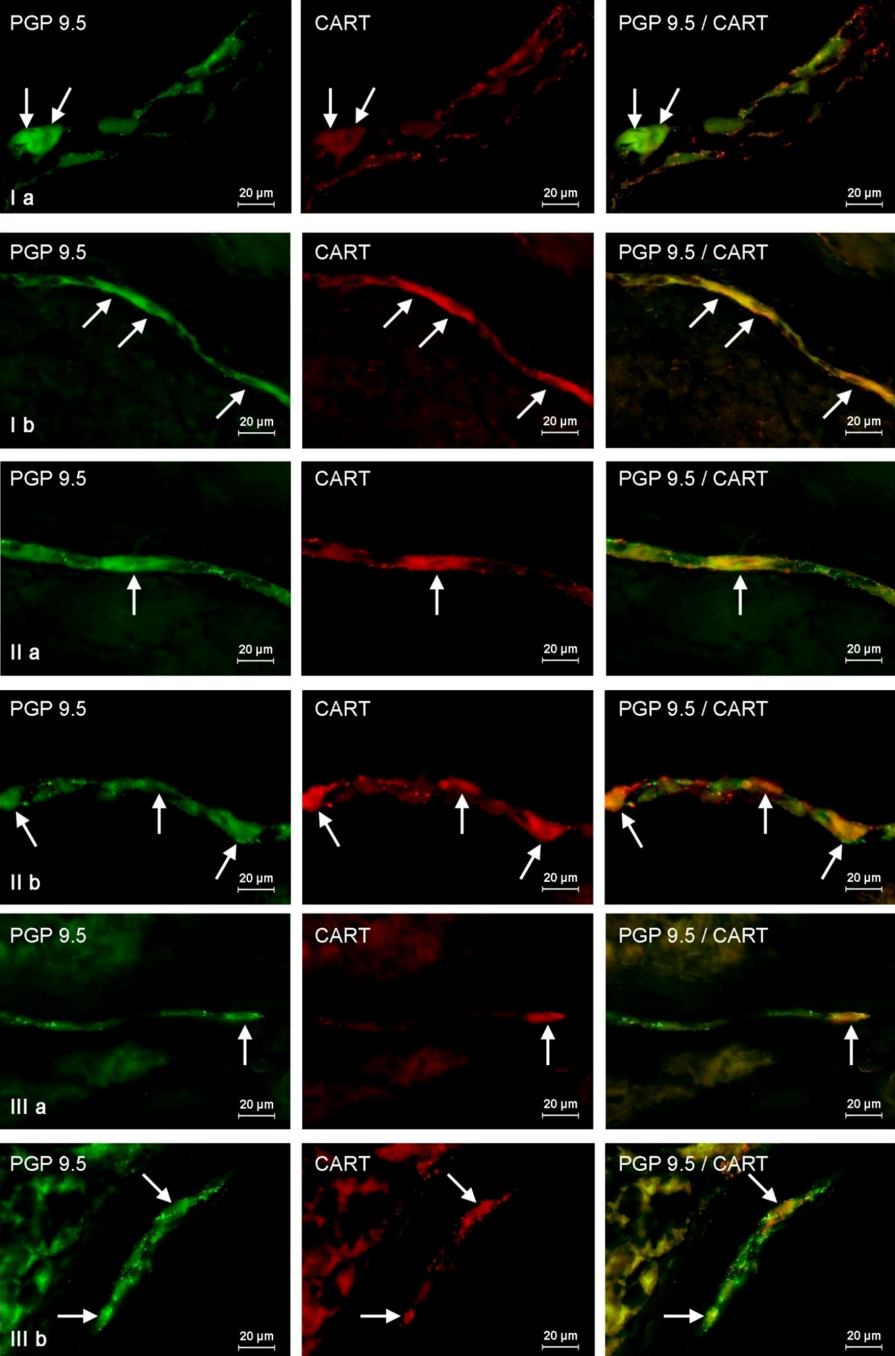
Distribution pattern of nervous structures immunoreactive to protein gene-product 9.5 (PGP 9.5)—used as pan neuronal marker and CART in the wall of porcine duodenum under physiological conditions (**a**) and after T-2 toxin administration (**b**); **I** myenteric plexus, **II** outer submucous plexus, **III** inner submucous plexus. CART-positive neurons are indicated by *arrows*. The *right column* of the pictures shows the overlap of both stainings

**Fig. 7:**
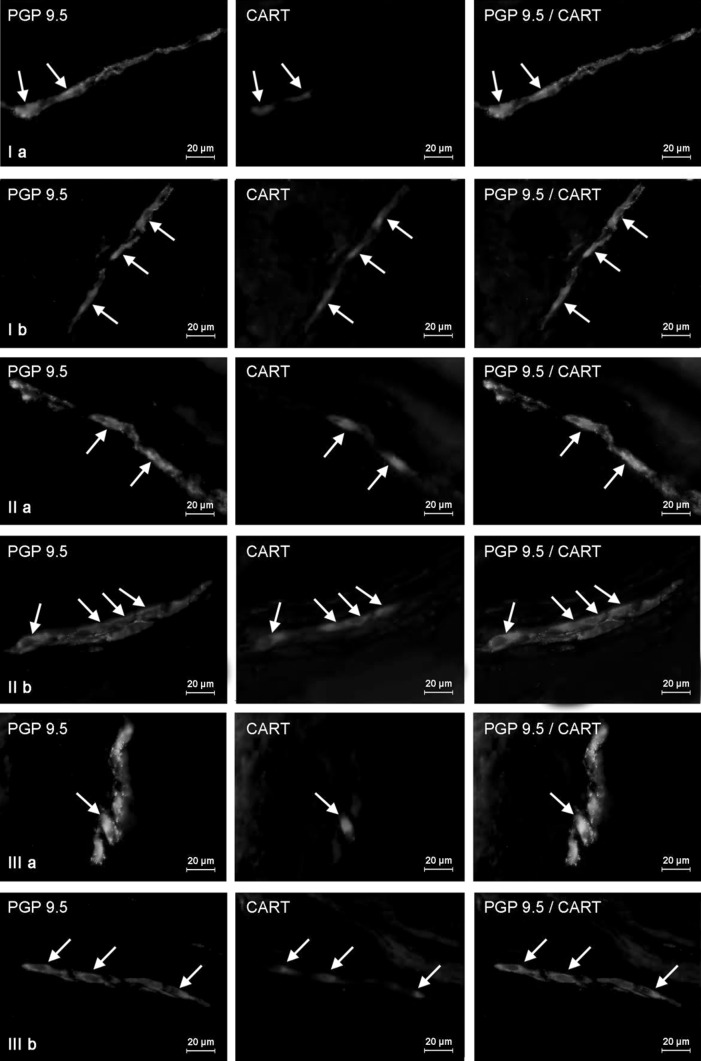
Distribution pattern of nervous structures immunoreactive to protein gene-product 9.5 (PGP 9.5)—used as pan neuronal marker and CART in the wall of porcine descending colon under physiological conditions (**a**) and after T-2 toxin administration (**b**); **I** myenteric plexus, **II** outer submucous plexus, **III** inner submucous plexus. CART-positive neurons are indicated by *arrows*. The *right column* of the pictures shows the overlap of both stainings

The higher number of CART-positive enteric neurons in intestinal fragment studied of control animals was observed (like in the stomach) within myenteric plexus (Table 1). Nonetheless, the percentage of these neurons was lower than in the stomach and amounted to 38.10 ± 3.43 % in duodenum (Fig. 6Ia) and 33.43 ± 3.39 % within descending colon (Fig. 7Ia). Moreover, in MP of both fragments of intestine a dense network (+++) of intraganglionic nerve processes immunoreactive to CART was noted. In turn, the number of CART-LI neuronal cells within submucosal plexus, which in the intestine is divided into outer and inner submucous plexuses, was higher than in the stomach. In duodenum, these values amounted to 28.70 ± 0.90 and 21.96 ± 1.85 % in the OSP and ISP, respectively (Fig. 6IIa, IIIa). In descending colon, the percentage of submucosal neurons immunoreactive to CART was similar and came to 27.50 ± 1.07 % in the OSP (Fig. 7IIa) and 19.07 ± 4.11 % in the ISP (Fig. 7IIIa). In addition, in both “types” of submucosal plexuses in duodenum, rare (++) CART-LI intraganglionic nerves have been observed. In descending colon, a network of CART-LI nerve processes within OSP was denser (++) than in the ISP, where only single such nerve fibers were investigated (+).

The number of intramuscular CART-positive nerve fibers was similar in both intestinal fragments studied and slightly higher than that observed in the stomach. It amounted to 15.99 ± 0.80 and 15.33 ± 1.77 in duodenum (Fig. 4IIa) and descending colon (Fig. 4IIIa), respectively. The number of CART-LI nerve fibers observed in the intestinal mucosal layer was also higher than in the stomach, but contrary to intramuscular nerves, differences between duodenum and descending colon were noted. Namely, in duodenum (Fig. 5IIa) this value amounted to 3.07 ± 0.14 of CART-LI nerves per observation field, whereas in descending colon—1.94 ± 0.35 (Fig. 5IIIa).

The administration of T-2 toxin changed the percentage of CART-LI enteric neurons, as well as the density of nerves immunoreactive to this peptide. Generally, these changes included a greater number of CART-LI nerve structures, but their intensity clearly depended on the fragment of gastrointestinal tract and part of the ENS studied (Table 1).

The most visible increase in the percentage of CART-positive neurons, about 25 percentage points (pp), have been noted in duodenal myenteric (Fig. 6Ib) and outer submucous plexuses (Fig. 6IIb), as well as in the ISP of descending colon (above 23 pp) (Fig. 7IIIb). Slightly lower changes were observed in the gastric MP (above 17 pp) (Fig. 3Ib), duodenal ISP (Fig. 6IIIb), as well as colonic MP and OSP (Fig. 7Ib, IIb) (almost 14 pp in the three mentioned plexuses). The smallest number of neurons immunoreactive to CART was investigated in submucosal plexus of the stomach, where changes amounted to about 7 pp. After T-2 toxin administration, like in control animals, two “kinds” of gastric submucous ganglia were noted. The majority of them were devoid of any CART-LI neurons (Fig. 3IIb), but also ganglia with high number of cells immunoreactive to CART were observed (Fig. 3IIb1). In T-2 group, density of intraganglionic CART-LI nerve fibers was also higher than in control group, but these changes were not very clear and were observed in the gastric MP, duodenal ISP, and all “kinds” of plexuses in descending colon (Table 1).

Moreover, clear changes in the density of muscular and mucosal CART-positive nerve fibers were observed after T-2 toxin administration. The most visible percentage increase was noted in duodenal muscular layer (above 10 pp) (Fig. 4IIb), whereas in other gastrointestinal fragments studied, these values amounted to almost 7 pp in the stomach (Fig. 4Ib) and about 8 pp in descending colon (Fig. 4IIIb). Changes in the number of CART-positive nerves in the mucosal layer (Fig. 5) were less visible and amounted to about 5 pp in duodenum and about 2 pp within stomach and descending colon.

## Discussion

The results obtained during the present investigation show that CART seems to be an important neuronal factor in the enteric nervous system of porcine stomach, small and large intestine. It is in agreement with previous studies, where CART has been described in the ENS of many species, including human (Ekblad 2006; Arciszewski et al. 2009; Gonkowski et al. 2012; Zacharko-Siembida and Arciszewski 2014). In the present experiment, the majority of CART-positive neuronal structures have been observed in myenteric plexus and circular muscle layer, what is also in agreement with previous studies (Ekblad 2006; Gonkowski et al. 2012; Bulc et al. 2015). On the other hand, during this study very few population of CART-LI neurons have been also noted within the gastric submucous plexus. This is a clear difference from previous studies, where neurons immunoreactive to CART have not been noted in this part of the ENS within the porcine stomach (Zacharko-Siembida and Arciszewski 2014; Rekawek et al. 2015). Although the presence of CART in gastric submucous plexus seems to be compatible with observation concerning the participation of this peptide in regulation of gastric acid secretion (Okumura et al. 2000; Janiuk et al. 2013), the reason of this disparity between previous studies and present experiment is unclear. Probably, gastric porcine submucosal neurons can produce CART under various physiological, nutritional, and/or farming factors. So, even slight differences in feed composition, farming conditions, and environmental bacterial flora can cause changes in neurochemical coding of enteric neurons. The influence of the above-mentioned factors on CART-like immunoreactivity in the ENS is more presumable, because the exact roles of this peptide in the gastrointestinal tract are still not fully explained. Previous studies described that CART can take part in mitigation of colon motility (Tebbe et al. 2004), reduction of gastric acid secretion, and regulation of stomach emptying (Okumura et al. 2000; Janiuk et al. 2013; Zacharko-Siembida and Arciszewski 2014), but mechanisms of these actions are unknown. Probably they are realized with the use of the central nervous system, where CART is relatively well-known factor taking part in feeding behavior (Risold et al. 2006), because direct action of CART on isolated fragments of the GI tract does not change contractile or relaxational activity of intestinal muscles (Ekblad et al. 2003). On the other hand, it is known that CART in the enteric nervous structures co-localizes with a wide range of neuronal active substances (Gonkowski et al. 2013; Bulc et al. 2014), which can suggest that this peptide plays multifold functions in intestinal regulatory processes.

Moreover, previous studies describe that some pathological factors can influence the population of CART-LI neurons within the ENS (Gunnarsdóttir et al. 2007; Gonkowski et al. 2012, 2015), which may suggest neuroprotective and adaptive roles of this peptide in the digestive system, as well as its participation in pro- or anti-inflammatory processes.

Also during the present study, the changes in CART-like immunoreactivity in the porcine ENS after T-2 toxin administration have been observed. On the one hand, this observations show that even low doses of T-2 toxin are not neutral for living organism and can cause changes in the enteric nervous system. On the other hand, they confirm that CART takes part in the pathological processes in the GI tract. It should be pointed out that both exact reasons and mechanisms of observed changes in CART-like immunoreactivity are not clear and may be a result of various factors.

Regarding the reasons of observed changes, they can be a result of direct action of T-2 toxin on neuronal cells, and the increase in CART-like immunoreactivity may be an adaptive process of neurons in response to the impact of this mycotoxin, especially the damage of mitochondria, which has been described in previous studies (Marin et al. 2013; De Ruyck et al. 2015). On the other hand, fluctuations of the number of CART-LI neuronal structures may be connected with indirect actions of T-2 toxin, namely with ATA—morbidity with various gastrointestinal symptoms, such as vomiting, diarrhea, or nausea (Lutsky and Mor 1981; De Ruyck et al. 2015). One cannot exclude that changes in actions of GI tract connected with the above-mentioned symptoms, as well as inflammatory processes accompanying ATA can be the reason of fluctuations in CART-like immunoreactivity in the ENS, especially that the influence of inflammation on chemical coding of enteric neurons is relatively well known (Vasina et al. 2006).

The mechanisms of the increase in CART-LI enteric neuronal cells number after T-2 toxin administration are also unclear. Observed changes can be a result an augmentation of CART synthesis within enteric neurons, as well as fluctuations in molecular transport of this peptide between cells bodies and nerve endings. Most probably, changes in CART-like immunoreactivity noted during the present study are caused by the increase in the synthesis of CART in neuronal cell bodies with simultaneous intensification of molecular transport of this peptide from perikarya to nerve endings. This hypothesis is supported by the fact that during the present investigation the increase in CART-like immunoreactivity has been observed both in enteric neuronal cell bodies and nerve fibers. In turn, fluctuations in synthesis of CART may arise from various changes in the transcription, translation, and/or post-translational modification, where pro-peptide under convertase enzymes transforms to biological active form of CART (Maixnerová et al. 2007).

## Conclusions

To sum up, the obtained results show that T-2 toxin in low doses may affect the enteric nervous system and therefore even suggested permissible doses in feed cannot be qualified as completely safe for living organisms, what has been suggested in previous studies (Obremski et al. 2008, 2013). Moreover, the present study confirms that CART seems to be an important neuronal factor within the enteric nervous system, which takes part in regulation of intestinal functions both in physiological conditions and during pathological states. Due to the fact that the increase in CART-like immunoreactivity has been observed during the present study as well as in previous investigations on zearalenone intoxication (Gonkowski et al. 2015), and it is well accepted that expression of neuroprotective substances usually increases under the majority of pathological stimuli (Arciszewski and Ekblad 2005), it can be assumed that this peptide also plays neuroprotective roles in the ENS. On the other hand, some previous studies on various pathological factors (such as axotomy or inflammatory processes) have shown the decline in CART-LI enteric neurons number (Burliński 2012; Gonkowski et al. 2012). So, the exact functions of CART in intestinal innervation still remain not fully explained and require further investigations.
